# Mechanisms of Phytochemicals in Anti-Inflammatory and Anti-Cancer

**DOI:** 10.3390/ijms24097863

**Published:** 2023-04-26

**Authors:** Ji-Hoon Jang, Tae-Jin Lee

**Affiliations:** Department of Anatomy, College of Medicine, Yeungnam University, 170 Hyeonchung-ro, Nam-gu, Daegu 42415, Republic of Korea; 21300447@yu.ac.kr

Phytochemicals are chemical compounds that exist in plants and serve various functions such as protecting against pests, UV radiation, and diseases. While these compounds are not essential nutrients for humans, they are known to have various physiological functions, such as antioxidant, anti-inflammatory, anti-cancer, anti-diabetic, and immune enhancement effects. Even now, various phytochemicals with potential as therapeutic candidates for cancer and inflammation are being discovered and studied.

Phytochemicals can regulate the expression of various oncogenic or tumor suppressor proteins and the activity of transcription factors such as Forkhead box O, nuclear erythroid 2-related factor 2 (Nrf2), and nuclear factor-κB (NF-κB) in cell signaling pathways. In addition, estrogen receptors, receptor tyrosine kinases, and mitogen-activated protein kinases (MAPKs) can be regulated by direct interactions with phytochemicals. Phytochemicals exhibit tumor-suppressing activities by modulating the gene expression related to signaling pathways at various stages of cancer initiation, progression, metastasis, and regulation of cell death. Recently, the capacity of phytochemicals on specific mechanisms such as promoter DNA methylation, histone modification, and microRNA-facilitated post-transcriptional modification has been gaining attention as a new field for understanding and controlling anti-cancer properties.

The Special Issue, titled “Mechanisms of Phytochemicals in Anti-inflammatory and Anti-cancer” of the International Journal of Molecular Sciences, contains a total of seven articles, including six original articles and one review article. These articles comprehensively analyze the mechanisms underlying the anti-inflammation and anti-cancer effects of various aspects by phytochemicals.

Trávníček et al. [1] found that the gold (I) complex with the plant hormone kinetin can regulate anti-cancer and anti-inflammatory effects and peroxisome-proliferator-activated receptor gamma (PPARγ) in various cancer cells (A2780, A2780R, PC-3, 22Rv1, and THP-1). Please change the sentence as follow; In this research, treatment with complex (1) modulated cell cycle arrest and apoptosis, and weakly disturbed the mitochondrial membrane potential (MMP) in ovarian cancer cells. Moreover, complex (1) treatment slightly increased PPARγ in acute monocytic leukemia. By contrast, complex (1) treatment did not alter NF-κB activity. Complex (1) had no anti-inflammatory effect, but exerted significant anti-cancer effects on cancer cell lines by affecting DNA damage/repair, cell division, mRNA processing/splicing, and intracellular signaling.

According to a report by Ahmed et al. [2], phytochemicals are important antioxidants with a wide range of physiological actions that reduce oxidative stress and prevent cell damage, contributing to cancer prevention. They also have various functions, such as cell cycle regulation and apoptosis. In addition, phytochemicals are useful in preventing and treating inflammatory diseases. Mechanisms by which phytochemicals act in cancer prevention and the regulation of inflammation include the regulation of cell signaling pathways including NF-κB, MAPK, signal transducer and activator of transcription 3 (STAT3), cyclooxygenase-2 (COX-2), 5-lipoxygenase (5-LOX), and phosphatidylinositol 3-kinases (PI3K)/Akt. Thus, the usefulness of phytochemicals in their ability to modulate signaling pathways is thought to be important in modulating the interrelationship between cancer and inflammation.

Li et al. [3] revealed the anti-cancer effect and action mechanism of the essential oil extracted from Plagiomnium acutum *T. Kop*. The Plagiomnium acutum *T. Kop* essential oil (PEO) inhibited the cell growth of various cancer cell lines, including MCF-7, U87, A549, and HepG2. In particular, PEO induced cell growth inhibition and apoptosis at low concentrations in HepG2 and A549 cells; this is the G1-phase arrest by the upregulation of p21^Cip1^ and p27^Kip1^ in the cell cycle. Additionally, PEO significantly inhibited ROS production. Furthermore, PEO-induced apoptosis occurred via the mitochondrial pathway, as evidenced through cytochrome *c* release, caspase-9 and -3 activations, the decreased expression of the anti-apoptotic protein Bcl-2, and increased expression of the pro-apoptotic protein Bax.

Cheng et al. [4] explained the mechanism by which aloe emodin (AE) and emodin (EMD) could inhibit breast cancer metastasis. A co-culture model of MCF-7 cancer cells and HUVEC showed that HUVEC promotes the malignant phenotype of cancer cells. AE and EMD inhibited adhesion, invasion, metastasis, and angiogenesis. EMD showed a more significant inhibitory effect on adhesion than AE, while AE exhibited a more pronounced inhibitory effect on invasion than EMD. In angiogenesis, AE inhibited VEGF, but EMD had no effect. Moreover, AE inhibited MMP-9, whereas EMD inhibited both MMP-2 and MMP-9. Furthermore, metabolic analysis in MCF-7 cells revealed 27 and 13 biomarkers for AE and EMD, respectively. These biomarkers were related to polyamine metabolism, methionine cycle, TCA cycle, glutathione metabolism, purine metabolism, and aspartate synthesis. The inhibitory effect of typical metabolites was significantly better with AE than with EMD. AE and EMD both inhibited breast cancer cell metastasis, but their pathways were different. These findings suggest the potential of AE and EMD as potential breast cancer metastasis inhibitors, offering a new strategic approach to breast cancer treatment.

Sitarek et al. [5] described the cell death induction by three substances derived from Plectranthus ornatus, halimane (11R*,13E)-11-acetoxyhalima-5,13-dien-15-oic acid (HAL), the labdane diterpenes 1α,6β-diacetoxy-8α, 13R*-epoxy-14-lab den-11-one (PLEC), and a forskolin-like 1:1 mixture of 1,6-di-O-acetylforskolin and 1,6-di-O-acetyl-9-deoxyforskolin (MRC). HAL, PLEC, and MRC inhibited cell growth, but HAL and PLEC, except for MRC, increased ROS production, and reduced MMP and mitochondrial copy numbers in both breast cancer MCF-7 cells and hypopharyngeal squamous cell carcinoma FaDu cells. Additionally, mitochondrial DNA damage increased with HAL and PLEC, except for MRC, while nuclear DNA damage was not affected by any of the substances. Furthermore, gene expression analysis showed that HAL and PLEC, but not MRC, increased the expression of Bax, Caspase-3, -8, -9, Apaf-1, and TP53 mRNA levels, and decreased the expression of Bcl-2 and Mcl-1. Apoptosis was increased in HAL and PLEC except for MRC in cancer cell lines, but showed no toxic effect in in vivo experiments.

Reimche et al. [6] reported on a study which investigated the effects of phenanthroindolizidine Alkaloids (PAs) isolated from Tylophora ovata on inflammation, spheroid growth, and invasion in triple-negative breast cancer (TNBC) cells. It was found that PAs can regulate NF-κB activity and affect cell survival. Compound (1), one of the PAs, showed better growth inhibitory effects than paclitaxel, and it also inhibited invasion in TNBC cells. Furthermore, compound (1) was able to reduce hypoxia-inducible factor 1-alpha activity, inhibit NF-κB activation through IκBα stabilization, and delay cell cycle progression. Further studies are needed to find the optimal conditions for drug application and to reduce neurotoxicity through combination treatment with paclitaxel.

The review by Sohn et al. [7] introduced the medical applications of phytosterols found in seaweeds. Fucosterol, which is most commonly found in brown seaweed, has potential as an antioxidant due to its ability to increase antioxidant enzymes such as superoxide dismutase, catalase, and heme oxygenase-1, as well as the Nrf2 transcriptional level, preventing the generation of ROS [8]. It also has the potential as an anti-inflammatory agent, as it inhibits the synthesis of nitric oxide (NO) and the expression of COX-2, which are inflammatory mediators induced by LPS [9]. According to other reports, fucosterol can inhibit TNF-α, IL-6, IL-1β, and NF-κB activity [10], and suppress the generation of pro-inflammatory molecules by the p38 MAPK activation [11]. It is also associated with Alzheimer’s disease, as it reduces the accumulation of toxic protein amyloid-β [12]. Stigmasterol has potential as an anti-cancer agent, as it increases Bax expression, decreases Bcl-2 expression, and inhibits the JAK/STAT signaling pathway in gastric cancer cell lines [13]. Campesterol increases the expression of cytochrome *c*, Bak, Bax, and autophagy-related proteins such as BECN1 in ovarian cancer cell lines [14]. Brassicasterol inhibits androgen receptor expression and activates caspase3 in prostate cancer cell lines [15]. The β-sitosterol induces cell cycle arrest while increasing Bax and decreasing Bcl-2 expression in colon and lung cancer cell lines [16,17]. This paper proposes the potential medical applications of phytosterols in seaweeds because they possess the following diverse biological activities: immune regulation, antioxidant, anti-inflammatory, and anti-cancer effects.

Overall, the Special Issue “Mechanisms of Phytochemicals in Anti-inflammatory and Anti-cancer” has the potential to provide valuable insights into the use of phytochemicals as natural substances for preventing and treating cancer and inflammation. Further research in this area may lead to a better understanding of the mechanisms of action of these compounds and their potential use in combination therapies. Moreover, these compounds could offer new options for preventing and treating cancer, thereby providing novel and effective therapies for cancer patients.
